# Effects of the Financial Crisis on Psychotropic Drug Consumption in a Cohort from a Semi-Urban Region in Catalonia, Spain

**DOI:** 10.1371/journal.pone.0148594

**Published:** 2016-02-12

**Authors:** Maria A. Barceló, Montserrat Coll-Negre, Gabriel Coll-de-Tuero, Marc Saez

**Affiliations:** 1 Research Group on Statistics, Econometrics and Health (GRECS), University of Girona, Girona, Spain; 2 CIBER of Epidemiology and Public Health (CIBERESP), Madrid, Spain; 3 Cambian Churchill Hospital, Mental Health Services, London, United Kingdom; 4 Research Support Unit, University Institute of Research in Primary Care Jordi Gol (IdIAP Gol), Girona, Spain; 5 Department of Medical Sciences, University of Girona, Girona, Spain; Yokohama City University, JAPAN

## Abstract

**Purpose:**

Evidence of whether the recent economic crisis has or has not had an effect on psychotropic drug consumption is very scarce. Our objective was to determine if there had in fact been an increase in psychotropic drug use as a result of the financial crisis.

**Methods:**

In our study a retrospective cohort (between January 1, 2005, and December 31, 2012) was made up of individuals from the general population in a region in the northeast of Catalonia, Spain. We specified a generalized linear mixed model along with combined ‘selection on observables’ as (propensity scoring) matching and ‘selection on unobservables’ as (random coefficient) the panel data model methods, and performed inferences using a Bayesian framework.

**Results:**

In the period following the economic crisis (post 2009), there was an increase in the consumption of psychotropic drugs which was significantly higher among those who had already been consuming psychotropic drugs prior to 2009 and those most likely to be unemployed. The increase was of greater significance when consumption was measured by the number of drugs being taken, rather than by the defined daily dose (DDD), with the greatest increase occurring in 2011; the very year in which Spain was most affected by the crisis.

**Conclusions:**

Once the financial crisis had ended, there was an increase in the severity, rather than the intensity, of mental health disorders in individuals who had already had disorders before the crisis. This increase occurred in those most likely to be unemployed, and the severity was accentuated in the toughest year of the economic crisis.

## Introduction

Mental health disorder is a global health issue which significantly affects not only the quality of life of the ill individuals and their families, but also leads to serious social consequences and economic costs for a country [1]. In Europe mental health disorders in general are the most common cause of disability and major depression ranks as the third most-common condition after ischemic heart disease and stroke [2], thus making mental health illness a priority in European Union health policy [3].

In Ruhm [4,5] at least, there is considerable evidence that (good) health is counter-cyclical, i.e. health outcomes improve with recessions. However, there is also considerable evidence that economic recessions themselves are a risk to the mental health of a population [6–14].

Recessions are defined as two consecutive quarters of negative economic growth measured by the seasonally adjusted quarter-on-quarter figures for real GDP [15]. In the U.S. the economic recession began in December 2007. Using three waves of a population-based panel study of US households for the period around the economic recession (2004 to 2009), McKenzie *et al*. showed that ecological measures of the effects of the financial crisis, such as changes in household income and in deprived areas, along with individual measures, such as moving from employment to inactivity and changes in individual deprivation, both lead to a short-term deterioration of (self-perceived) mental health [12]. However, individual measures illustrated a much greater effect. A study in Canada, conducted during the early years of the economic recession, January 2008-October 2009, (the economic recession for Canada also began in December 2007), showed an increase in the prevalence of major depression, but did not detect any change in generalized anxiety disorder, panic disorder or social phobia [13]. In Spain, a study comparing patients attending primary healthcare centres in 2006–2007 and 2010–2011 (the Spanish economy went into recession in the first quarter of 2009) showed significant increases not only in depression, but also in other mental health disorders including anxiety, somatoform disorders and problems related to alcohol consumption [10,11]. The authors attribute a third of the risk of mental health disorders to job loss and mortgage difficulties. Using population-based surveys involving approximately one million respondents aged 25 or older from the United States for 2003–2010, Nandi *et al*. found that individual-level unemployment was associated with a deterioration in health behaviours (higher levels of obesity, smoking and alcohol consumption) [14]. However, on a contextual level, changes in the (local-area) unemployment rate were not consistently associated with those same health behaviours. Using cross-sectional data from the Spanish Health Survey for the years 2006 and 2011–2012, Urbanos-Garrido and López-Valcárcel found that unemployment had an impact on both (self-perceived) mental health and (self-perceived) overall health [16]. They also found that the negative effects on mental health were exacerbated significantly during the economic crisis. Conversely, Astell-Burt and Feng [17], using a UK labour force survey repeated every three months from 2006 to 2010 (in the UK the economic recession began in the second quarter of 2008), showed that while there was an increase in (self-perceived) cardiovascular and respiratory problems this did not occur for mental health disorders, including depression. However, they did specify that responses to the mental health question in the survey were not rigorous and petitioned to have K10 or GHQ scores incorporated in the following waves to obtain reliable results in relation to mental health.

Furthermore, in the European Union the increase in intentional and violent premature deaths, mainly suicides [6,18–21], self-harm [19] and suicide attempts [22], all of which could be considered part of the spectrum of mental disorders, have been associated with the current economic recession.

During a recession, as a result of a perceived reduction in income and investments and the fear of job loss, a perception of poorer mental health is observed not only among the unemployed, but also the employed; especially amongst manual workers or workers with routine activities [7,16,17]. Since psychotropic drugs (antidepressant and anxiolytics) mitigate many of the effects of mental health disorders, then the rationale would be that a greater perceived risk by the population [23] should lead to an increased use of these drugs. Thus, for example, the Nuffield Trust and Health Foundation’s QualityWatch research programme [24] stated in its report that since the beginning of the financial crisis in the UK, the number of antidepressants being dispensed annually had risen to 8.5% compared with 6.7% before [25]. However, an increase in depression (0.8% increase between 2009–2010 and 2011–2012) could not fully explain the upsurge in the prescription of antidepressants [25]. During the period 2000–2011 [23,26], an increase in the use of anxiolytics and hypnotics was reported in Spain^c^, while for the period 2000–2012 there was an increase in the use of antidepressant drugs [10,11,27]. It should be noted that although these increases are greater than those produced in other European countries over the same time period [26], the average consumption of anxiolytics is higher in Spain than in other European countries [29]. However, in the cases of antidepressants and antipsychotics, these are clearly below the European average [29]. It is also worth noting that while the sales of antidepressants continued to increase during the economic crisis, (10% rise between 2009 and 2012) [11], the consumption of antipsychotics between 2009 and 2012 remained practically unchanged. Sales of hypnotics and anxiolytics, despite seeing an increase between 2009 and 2010 (3.6% and 4.5%, respectively) declined in 2011 and again in 2012 [11].

Our objective is to determine whether the current economic recession has resulted in an increase in the consumption of prescribed psychotropic drugs. To meet this objective, we used a cohort (i.e. a panel of data on an individual level) from a region in the northeast of Catalonia, Spain covering a period from 2005 to 2012.

## Materials and Methods

### Data setting

We used a retrospective, general population cohort, composed of individuals who, between January 1, 2005 and December 31, 2012, had made use of the primary healthcare services offered by one of the three Basic Areas of Health (ABS, *‘Àrees Bàsiques de Salut’*, acronym in Catalan) primary healthcare centres which are managed by the Institute of Health Care (IAS, *‘Institut d’Assistència Sanitària’* in Catalan).

The Catalan public healthcare system guarantees universal and free healthcare to all its citizens. This system is characterized by a division between healthcare funding (from the Catalan public budget) and the provision and management of the healthcare services. A user can decide to choose the public system or private (or mixed) healthcare. The IAS is a primary healthcare service provider in a mixed, public-private partnership. Catalonia is divided into seven health regions of which an ABS is a territorial division. All residents in an area covered by an ABS are ‘assigned’ to the provider responsible for that particular ABS [30]. That is to say, all of them will therefore have the same provider, who will likewise be the one managing the ABS. Each individual can choose both their health professional and centre from those available in the ABS.

The IAS manages all the ABSs that provide healthcare to the region of ‘La Selva Interior’, Girona (ABS Anglès; ABS Breda-Hostalric; and ABS Cassà de la Selva). *La Selva Interior* and *La Selva Marítima* form the ‘*comarca’* of La Selva, A ‘*comarca’* could be considered equivalent to a county. According to the Catalan Institute of Statistics (IDESCAT, *‘Institut d’Estadística de Catalunya’*), in 2012 the region’s population constituted 32,860 men and 32,702 women (0.87% and 0.85%, respectively, of the entire Catalan population). The area is defined as a mainly rural (or semi-urban) territory. There are a number of towns scattered throughout the area and it is also dispersed with farms, houses and small somewhat isolated villages. There are 144 municipalities in the region (representing 3.70% of Catalonia), but only five municipalities have more than 5000 inhabitants and only one has a little over 10,000. In 2012, the population density median was 85.5 hab/km^2^ and the average population density was 176.2 hab/km^2^ (compared to 235.8 hab/km^2^ in the whole of Catalonia [31].

In the study period, 25,943 men (78.95% of the men assigned to the ABS) and 25,414 women (77.71% of the women assigned) paid at least one visit to healthcare services (2.24 visits per month on average in the case of men, standard deviation (sd) equal to 1.99, median 2, first quartile (Q1) 1, third quartile (Q3) 3; 2.29 visits per month in the case of women, sd 2.00, median 2, Q1 1, Q3 3). In this study, while we focused on adult users aged 15 years or older (of which there were 42,267 (82.3%)), we were only interested in those who had used a psychotropic drug in the 2005–2012 study period.

All the data were obtained from clinical records and stored following a standardized protocol in the centralized IAS information system. The data for this study were drawn from that information system conforming to an anonimized clinical-administrative database.

The psychotropic drugs considered in this study were anxiolytics (benzodiazepines) and antidepressants. Anxiolytics included alprazolam, clorazepate, clonazepam, diazepam and lorazepam, and antidepressants included both selective serotonin reuptake inhibitors (citalopram, escitalopram, fluoxetine, paroxetine and sertraline) and serotonin-norepinephrine reuptake inhibitors (venlafaxine).

In the Catalan health system, psychotropic drugs must be prescribed by a physician and can not be purchased over the counter. In our study, we have included all prescriptions prescribed by a physician working in the public system, be they a psychiatrist or not. Although a patient could have been prescribed psychotropic drugs by a physician in private practice, they would then, and only then, be able to have that prescription filled if they asked their public health practitioner or family doctor to issue a corresponding electronic prescription. Therefore, all psychotropic prescriptions in the region, have been covered by our study.

To assess the consumption of psychotropic drugs we considered two indicators: number of drugs prescribed per individual/month and defined daily dose (DDD) per individual per month.

For each drug, we calculated the DDD per individual/month as follows:

$$DDD\;per\;individual/day=\frac{Number\;of\;packs\;\;x\;Units\;per\;pack\;x\;Amount\;of\;active\;subs\;\text{tan}\;ce}{DDD_{ATC}}$$

where DDD_ATC_ denotes the defined daily doses of the active substance, according to the definition from the World Health Organization’s (WHO) Anatomical Therapeutic Chemical classification system (ATC) DDD index for 2104 [32].

Next, we aggregated this measure for each individual and month to obtain the DDD per individual/month.

### Statistical methods

In order to estimate the monthly consumption of the prescribed psychotropic drugs (per individual), we specified a generalized linear model (GLM) for two dependent variables: number of drugs (per individual/month) and DDD (per individual/month):

$$E\left(Y_{it}\right)=\mu_{it}\;\mu_{it}=\text{log}\left(\eta_{it}\right)\;Var\left(Y_{it}\right)=\phi \mu_{it}$$ (1)

where the subscript i denotes individual and t month (from January 2005 to December 2012, t = 1,2,…,96), Y one of the two dependent variables (number of drugs and DDD) and μ the (conditional) mean.

While the number of drugs was countable, the DDD was a continuous variable. Thus, the number of drugs was distributed as a Poisson variable and, therefore, the log and the identity were, respectively, the appropriate link and variance functions. Since DDD was distributed as a Gaussian variable, we used identity for both link and variance functions. In both cases ϕ was a dispersion parameter, allowing the observed variance to be higher than the theoretical variance (i.e. equal to the mean when the variable is distributed as a Poisson variable or equal to a constant when the variable is distributed as a Gaussian variable).

The economic recession could be considered a ‘natural experiment’ since (1) intervention was not undertaken for research purposes and (2) variation in exposure and outcomes was analysed using methods that attempt to make causal inferences [33]. The methods that study natural experiments have the same validity threats as those of experimental methods (i.e. randomized controlled trials). The main difference is the absence of randomization. In fact, in a natural experiment study there is no general solution for the presence of selection bias, i.e. the problem of confounding. As a consequence, all natural experiment studies require a comparative group (i.e. control group or ‘counterfactual’) to provide an indication of what would have happened in the absence of intervention [34].

We used individuals in the cohort who had not taken any kind of psychotropic drug prior to 2009 (the beginning of the economic crisis) as our control group. This encompassed 13.2% of the individuals in the cohort. Since the number of covariates was very high, exact matching was impracticable in our case so we used propensity scoring [35] to match the method. That is to say, instead of trying to ensure that the control (an individual who was not a consumer before 2009) matched each case (an individual who was a consumer before 2009) and had exactly the same values for the variables used for matching, matching was based on the ‘probability of being’. Specifically we used the following procedure:

1. We estimated a logistic regression with a dependent variable and conditioning variables, which were the same as we included in the linear predictor (η in equation {1}). Thus, we obtained the propensity score, i.e. the predicted ‘probability of being’ case.
2. Next, we matched each case to controls on propensity.
3. Finally, we used propensity scoring stratification.

Rosenbaum and Rubin [34] pointed out that stratification based on propensity scoring produces strata in which the effect estimated in each stratum is an unbiased estimator of the true treatment effect.

In the additive linear predictor η in {1} we introduced the variables that would be able to explain the consumption of psychotropic drugs:

$$\begin{array}{l} \eta_{it}=\beta_{0i}+\alpha_{ti}+\\ \;\beta_{1}Sex_{i}+\sum_{k=2}^{6}\beta_{k}AgeGroup_{k,it}+\sum_{k=1}^{7}\beta_{6+k}Country_{k,i}+\\ \;\beta_{15}MS_{it}+\sum_{k=1}^{2}\beta_{15+k}Cancer_{k,it}+\sum_{k=2}^{5}\beta_{16+k}Qu\;\text{int}\;ile\_Unempl_{k,it}+\beta_{21}Visits_{it} \end{array}$$

In particular we included explanatory variables, namely, gender (with men as the reference category), age group (15–34 years–reference category -, 35–44 years, 45–54, 55–64, 65–74, 75 and older), country (country of birth, 0 Spain–reference category -, 1 European Union–with the exception of countries from the former Eastern Bloc -; 2 Eastern Europe; 3 Maghreb countries; 4 Latin America; 5 Sub-Saharan Africa; 6 India-Pakistan; 7 other Asian countries; 8 other OECD countries), the presence of metabolic syndrome (MS); cancer (1 any neoplasm; 2 fear of having cancer; 0 otherwise–reference category), the quintiles of the probability of being unemployed (and also of being unemployed for over a year) (Quintile_Unempl), and the monthly number of visits to a physician (Visits). In order to control the time trend α was a time effect (constructed from t, t = 1…96) and β denotes unknown parameters associated to explanatory variables whose antilog was the relative risk associated to such variables.

According to the US National Cholesterol Education Program (NCEP) Adult Treatment Panel III (NCEP ATP III), metabolic syndrome (MS) is defined as a co-occurrence of three out of five of the following medical conditions [36,37]: hypertension, diabetes mellitus type II or elevated fasting plasma glucose, obesity, high serum triglycerides, and/or low HDL levels. For the definition of hypertension and diabetes mellitus type II, we used diagnoses by one of the physicians from the IAS. With respect to the other medical conditions that define MS, obesity was defined as Body Mass Index > 30 kg/m^2^ (height and weight were measured in the physician’s office). Elevated fasting plasma glucose was defined within the range of 101 mg/dL to 126 mg/dL, low HDL < 40 mg/dL for men and HDL < 50 mg/dL for women, hypertriglyceridemia triglycerides ≥150 mg/dL and hypercholesterolemia total cholesterol ≥200 mg/dL. Glucose, HDL, triglycerides and total cholesterol data were obtained from laboratory analyses.

There is some evidence that not only having cancer [38], but also the fear of having cancer (both conditions diagnosed by a physician) may affect the use of psychotropic drugs. Along these lines, women with false-positive results in mammography screening were reported as showing increased anxiety at recall vs. before screening, but this decreased six months after screening [39]. In addition, cancer survivors use more psychotropic drugs than those who have not had cancer [40].

Healthcare use in general and drug use in particular, besides being modulated by sex and age, is related not only to need variables (which ought to affect use), herein approximated by the metabolic syndrome (and partially by the visits to a physician), but also to non-need variables (which ought not to affect use) [41,42]. Therefore, we included explanatory variables in the models, i.e. the probability of being unemployed (and unemployed for over a year) and visits to a physician (in this case, it is only a partial proxy of a non-need variable). The probability of being unemployed (and unemployed for over a year) was then estimated (see details in S1 Appendix. Method of the estimation of the probability of an individual being unemployed).

Other variables such as history of suicide attempts, family size, presence of substance abuse or a family member with psychiatric illness, could have been included in the linear predictor but were not as these variables are not registered in the primary care clinical records, either because they are related to other health services (e.g. mental health services) or because they are not associated with individual’s variables, but rather their family history.

Note that, with the exception of gender and country of birth, all the explanatory variables were time-varying. In addition to age, individuals can enter the hypertensive status and/or diabetes status, and may enter or leave the status of obese and dyslipidemic, etc., and therefore MS. Furthermore, the likelihood of being unemployed and the number of visits to a physician varied during the study period.

Note also that, some of the coefficients had subscripts. In fact, we specified random coefficient panel data models. In mixed models terminology, we allowed (some of the) coefficients to be random effects [43], i.e. to be different for the various levels we considered. Thus, we allowed the intercept to be different for each individual, β_0i_, capturing characteristic individual specifics not already included in the model (i.e. unobserved individual heterogeneity). In this case we assumed that random effects were identical and independent Gaussian random variables with constant variance. The time effect varied by month and by individual, α_ti_, that is to say, we accepted that the time trend may not be linear (variation per month) and that the temporal effect may be different for each individual. We allowed, therefore, the slope to vary over time, thus enabling the effects of the recession to also be able to vary. As such, we assumed a random walk of order 1 (i.e. independent increments) for the Gaussian random effects vector (although we also assumed a constant variance) [44].

To conclude, we combined ‘selection on observables’, such as (propensity scoring) matching, and ‘selection on unobservables’ such as (random coefficient) panel data model methods. Jones and Rice show that panel data models with repeated individual data (i.e. the cohort we used) are, in fact, equivalent to the ‘difference-in-difference’ (DD) (the most commonly used method for ‘selection on unobservables’) [35]. One advantage of combining the DD approach with matching is that when there are unobservable variables inference will be contaminated with omitted variable bias through a failure to control them [35] and, therefore, the combination of the two methods is superior to pure (cross-sectional) matching estimators [45].

Given the complexity of our model, we preferred to perform inferences using a Bayesian framework. This approach is considered the most suitable to account for model uncertainty, both in the parameters and in the specification of the models. Moreover, only under the Bayesian approach is it possible to model extra variability (not captured by the Poisson link), with relatively sparse data in some cases. Finally, within the Bayesian approach specifying a hierarchical structure on the (observable) data and (unobservable) parameters, which are all considered as random quantities, is straightforward. In particular, we followed the Integrated Nested Laplace Approximation (INLA) approach [46], within a (pure) Bayesian framework. All analyses were carried out with the free software R (version 3.0.2) [47], through the INLA library [48,46].

### Ethics statement

Our retrospective study was approved by the Healthcare Institute of Girona’s Ethics Committee for Clinical Research (CEIC-IAS). As this was a retrospective study written informed consent was not obtained, however, any information that could have served to identify patients was anonymized and de-identified prior to analysis.

## Results

Among users aged 15 or over, 11,433 individuals (27.05%) took psychotropic drugs during the period under study or, more specifically, 4362 men (16.82%) and 7071 (27.82%) women.

The anxiolytics taken by individuals in the period 2005–2012 included alprazolam (used by 45.74%), diazepam (31.19%), lorazepam (19.16%), clorazepate (10.11%), and clonazepam (3.03%). In the case of antidepressants, 15.99% of the individuals took paroxetine, 8.87% escitalopram, 3.59% citalopram, 3.06% sertraline, 2.97% fluoxetine and 0.85% used venlafaxine.

Table 1 shows the composition of the cohort for the entire period under study, along with the sub-periods 2005–2008 and 2009–2012. Apart from the age of the individuals, there was little change in the cohort’s composition between the two sub-periods. When 2009–2012 is compared to the 2005–2008 sub-period, a slight increase in the percentage of foreigners (mainly Latin Americans) becomes apparent and, in particular, in the percentage of individuals located in the fifth quintile of the likelihood of being unemployed (all unemployed and long-term unemployed), which can clearly be attributed to the economic recession that began in 2009.

**Table 1:** Characteristics of the cohort.

	2005–2012	2005–2008	2009–2012	p-value
**Number of individuals**	11,433	7,413	7,322	9,398
**Age (years)**^1^	54.37 (17.72)	52.65 (17.23)	55.77 (17.98)	<0.001
**Percentage of women**	66.6%	67.1%	66.2%	0.313
**Metabolic Syndrome (%)**	1.6%	1.6%	1.6%	0.873
**Family in the cohort (%)**	20.2%	20.4%	20.1%	0.557
**Country of birth**	---	---	---	0.074
** Spain**	92.8%	93,9%	92,0%	---
** Maghreb**	2.4%	2.2%	2,6%	---
** Latin America**	2.0%	1.5%	2.4%	---
** European Union**^2^	1.1%	1.0%	1.1%	---
** East Europe**^3^	0.7%	0.5%	0.7%	---
** India-Pakistan**	0.7%	0.5%	0.8%	---
** Sub-Saharan Africa**	0.3%	0.3%	0.4%	---
** Other OECD**	0.1%	0.1%	0.1%	---
**Probability of being unemployed**^4^	---	---	---	<0.001
** 1**^**st**^ **Quintile**	7.5%	5.7%	8.9%	---
** 2**^**nd**^ **Quintile**	10.0%	9.2%	10.9%	---
** 3**^**rd**^ **Quintile**	12.3%	11.2%	13.1%	---
** 4**^**th**^ **Quintile**	17,8%	17.3%	18.2%	---
** 5**^**th**^ **Quintile**	23.3%	19.0%	28.6%	---
**Probability of being unemployed (long term)**	---	---	---	-<0.001
** 1**^**st**^ **Quintile**	5.1%	5.0%	5.2%	---
** 2**^**nd**^ **Quintile**	10.2%	10.1%	10.3%	---
** 3**^**rd**^ **Quintile**	13.6%	13.3%	13.9%	---
** 4**^**th**^ **Quintile**	16.1%	15.0%	17.0%	---
** 5**^**th**^ **Quintile**	25.9%	22.5%	30.0%	---

^1^ Mean (standard deviation)

^2^ With the exception of Romania

^3^ Including Romania

^4^ Percentage of individuals in the cohort. From least likely (first quintile) to most likely (fifth quintile) of being unemployed

**Fig 1:**
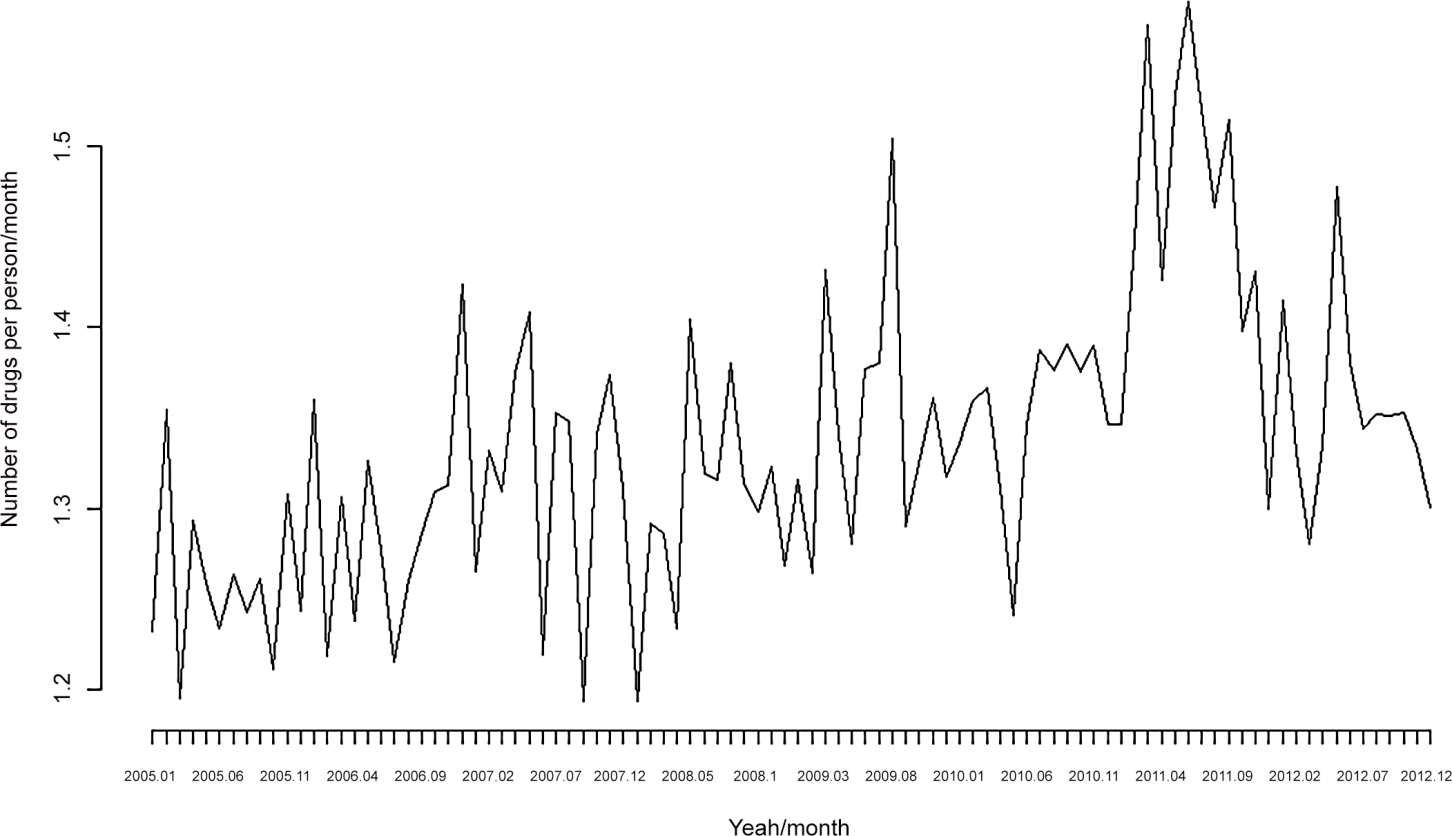
Consumption of psychotropic drugs. Number of drugs per person/month. Monthly data for the period 2005–2012.

**Fig 2:**
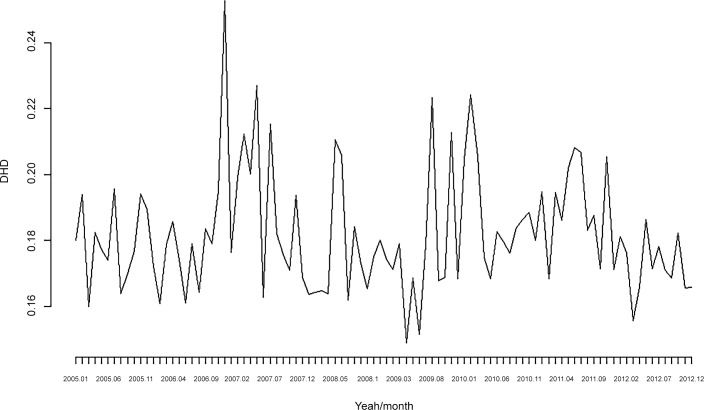
Consumption of psychotropic drugs. DDD per individual/month. Monthly data for the period 2005–2012.

The consumption of psychotropic drugs (without stratifying) is shown in Tables 2 and 3 and Fig 1 and Fig 2. Although it was not our objective, it is worth noting that the number of drugs per individual/month was higher in men than in women and that the number of drugs consumed did not follow a systematic behaviour between age groups (Table 2). However, the DDD per individual/month was much higher for women and there was also a systematic increase in DDD together with the age of the individual (Table 3). Furthermore, individuals located in the upper quintiles of the probability of being unemployed (both all and long-term unemployed) consumed more psychotropic drugs, both in terms of the number of drugs as well as the DDD.

**Table 2:** Consumption of psychotropic drugs. Number of drugs per individual/month.

Number of drugs per individual/month				
	2005–2012	2005–2008	2009–2012	p-value
**All**	1.180 (0.386)	1.162 (0.372)	1.194 (0.396)	<0.001
** Not consumers before 2009**	1.188 (0.429)	---	1.188 (0.429)	---
** Always consumers**	1.178 (0.381)	1.162 (0.372)	1.194 (0.390)	<0.001
**Sex**				
** Men**	1.193 (0.409)	1.174 (0.380)	1.209 (0.431)	0.012
** Women**	1.173 (0.373)	1.156 (0.368)	1.187 (0.377)	<0.001
**Age group**				
** 15–34 years**	1.183 (0.416)	1.166 (0.403)	1.201 (0.429)	0.095
** 35–44 years**	1.188 (0.420)	1.184 (0.400)	1.192 (0.441)	0.667
** 45–54 years**	1.185 (0.388)	1.165 (0.373)	1.202 (0.399)	0.037
** 55–64 years**	1.174 (0.376)	1.149 (0.334)	1.195 (0.406)	0.007
** 65–74 years**	1.162 (0.332)	1.143 (0.320)	1.190 (0.342)	0.027
** 75 years or older**	1.183 (0.376)	1.137 (0.327)	1.208 (0.398)	<0.001
**Probability of being unemployed**^1^				
** 1**^**st**^ **Quintile**	1.157 (0.403)	1.187 (0.427)	1.411 (0.390)	0.126
** 2**^**nd**^ **Quintile**	1.164 (0.375)	1.163 (0.379)	1.165 (0.373)	0.913
** 3**^**rd**^ **Quintile**	1.189 (0.406)	1.179 (0.373)	1.200 (0.435)	0.388
** 4**^**th**^ **Quintile**	1.187 (0.397)	1.162 (0.400)	1.206 (0.393)	0.015
** 5**^**th**^ **Quintile**	1.195 (0.400)	1.168 (0.383)	1.227 (0.418)	<0.001
**Probability of being unemployed (long term)**^1^				
** 1**^**st**^ **Quintile**	1.151 (0.387)	1.195 (0.414)	1.114 (0.360)	0.015
** 2**^**nd**^ **Quintile**	1.172 (0.384)	1.179 (0.395)	1.166 (0.376)	0.535
** 3**^**rd**^ **Quintile**	1.167 (0.386)	1.155 (0.361)	1.178 (0.406)	0.321
** 4**^**th**^ **Quintile**	1.191 (0.410)	1.170 (0.416)	1.206 (0.405)	0.073
** 5**^**th**^ **Quintile**	1.195 (0.399)	1.164 (0.376)	1.229 (0.420)	<0.001

^1^ From least likely (first quintile) to most likely (fifth quintile) of being unemployed

Values represent mean (standard deviation)

**Table 3:** Consumption of psychotropic drugs. DDD per individual/month.

DDD per individual/month				
	2005–2012	2005–2008	2009–2012	p-value
**All**	0.215 (0.603)	0.177 (0.470)	0.246 (0.690)	<0.001
** Not consumers before 2009**	0.132 (0.333)	---	0.132 (0.333)	---
** Always consumers**	0.223 (0.622)	0.177 (0.470)	0.268 (0.737)	<0.001
**Sex**				
** Men**	0.179 (0.613)	0.140 (0.376)	0.209 (0.747)	0.001
** Women**	0.234 (0.596)	0.196 (0.510)	0.265 (0.657)	<0.001
**Age group**				
** 15–34 years**	0.162 (0.780)	0.150 (0.538)	0.175 (0.970)	0.536
** 35–44 years**	0.173 (0.387)	0.154 (0.317)	0.189 (0.440)	0.060
** 45–54 years**	0.199 (0.473)	0.176 (0.455)	0.218 (0.486)	0.053
** 55–64 years**	0.245 (0.671)	0.212 (0.640)	0.272 (0.694)	0.059
** 65–74 years**	0.235 (0.606)	0.198 (0.467)	0.266 (0.698)	0.027
** 75 years or older**	0.263 (0.643)	0.172 (0.272)	0.322 (0.767)	<0.001
**Probability of being unemployed**^1^				
** 1**^**st**^ **Quintile**	0.109 (0.424)	0.121 (0.662)	0.104 (0.217)	0.594
** 2**^**nd**^ **Quintile**	0.136 (0.291)	0.143 (0.302)	0.132 (0.284)	0.504
** 3**^**rd**^ **Quintile**	0.148 (0.301)	0.147 (0.290)	0.149 (0.312)	0.940
** 4**^**th**^ **Quintile**	0.244 (0.905)	0.192 (0.675)	0.283 (1.044)	0.031
** 5**^**th**^ **Quintile**	0.266 (0.596)	0.211 (0.476)	0.333 (0.709)	<0.001
**Probability of being unemployed (long term)**^1^				
** 1**^**st**^ **Quintile**	0.094 (0.473)	0.124 (0.684)	0.069 (0.132)	0.172
** 2**^**nd**^ **Quintile**	0.136 (0.280)	0.146 (0.294)	0.128 (0.268)	0.238
** 3**^**rd**^ **Quintile**	0.149 (0.327)	0.130 (0.281)	0.165 (0.361)	0.088
** 4**^**th**^ **Quintile**	0.241 (0.897)	0.212 (0.724)	0.262 (1.001)	0.254
** 5**^**th**^ **Quintile**	0.262 (0.619)	0.205 (0.468)	0.323 (0.743)	<0.001

1 From least likely (first quintile) to most likely (fifth quintile) of being unemployed

Values represent mean (standard deviation)

On comparing the 2005–2008 and 2009–2012 sub-periods, (statistically significant) psychotropic drug consumption increased in the second sub-period (Tables 2 and 3). Note, however, that the increase seems to have been much more evident in the number of drugs (Fig 1) than in the DDD (Fig 2). This statistically significant increase in consumption was found for both men and women. Although a significant increase in consumption for all age groups can also be seen, this was only statistically significant (at 95%) in the number of psychotropic drugs from 45 year old group (Table 2) and the 65 year old group in the DDD (Table 3). With the quintiles of the likelihood of being unemployed, there was a statistically significant increase (with p<0.05) in the fourth and fifth quintile of the probability of being unemployed for both the number of drugs and the DDD, although in the case of the probability of being in long-term unemployment, the increase was only statistically significant (with p<0.05) for the fifth quintile.

**Fig 3:**
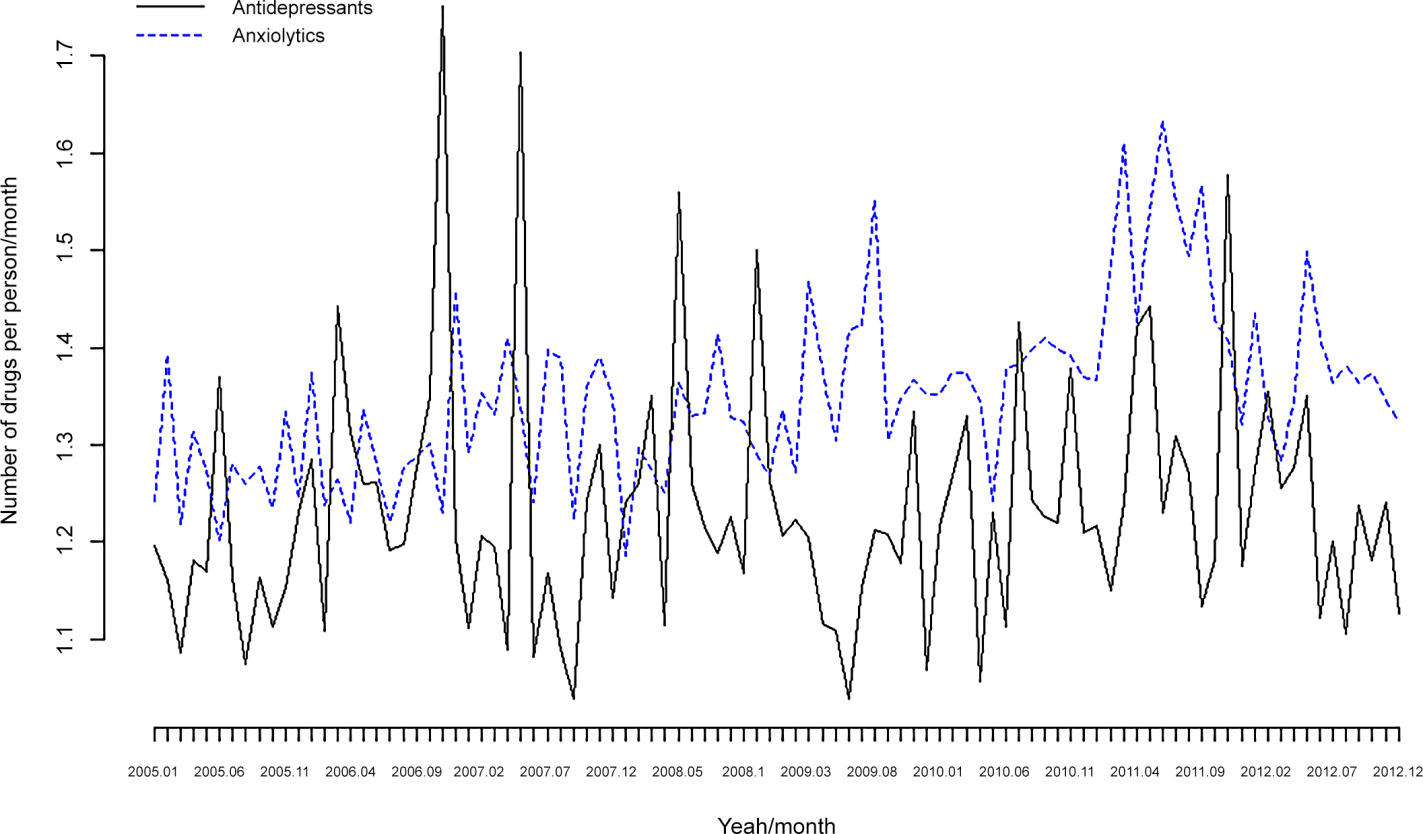
Consumption of psychotropic drugs. Antidepressants and anxiolytics. Number of drugs per person/month. Monthly data for the period 2005–2012.

**Fig 4:**
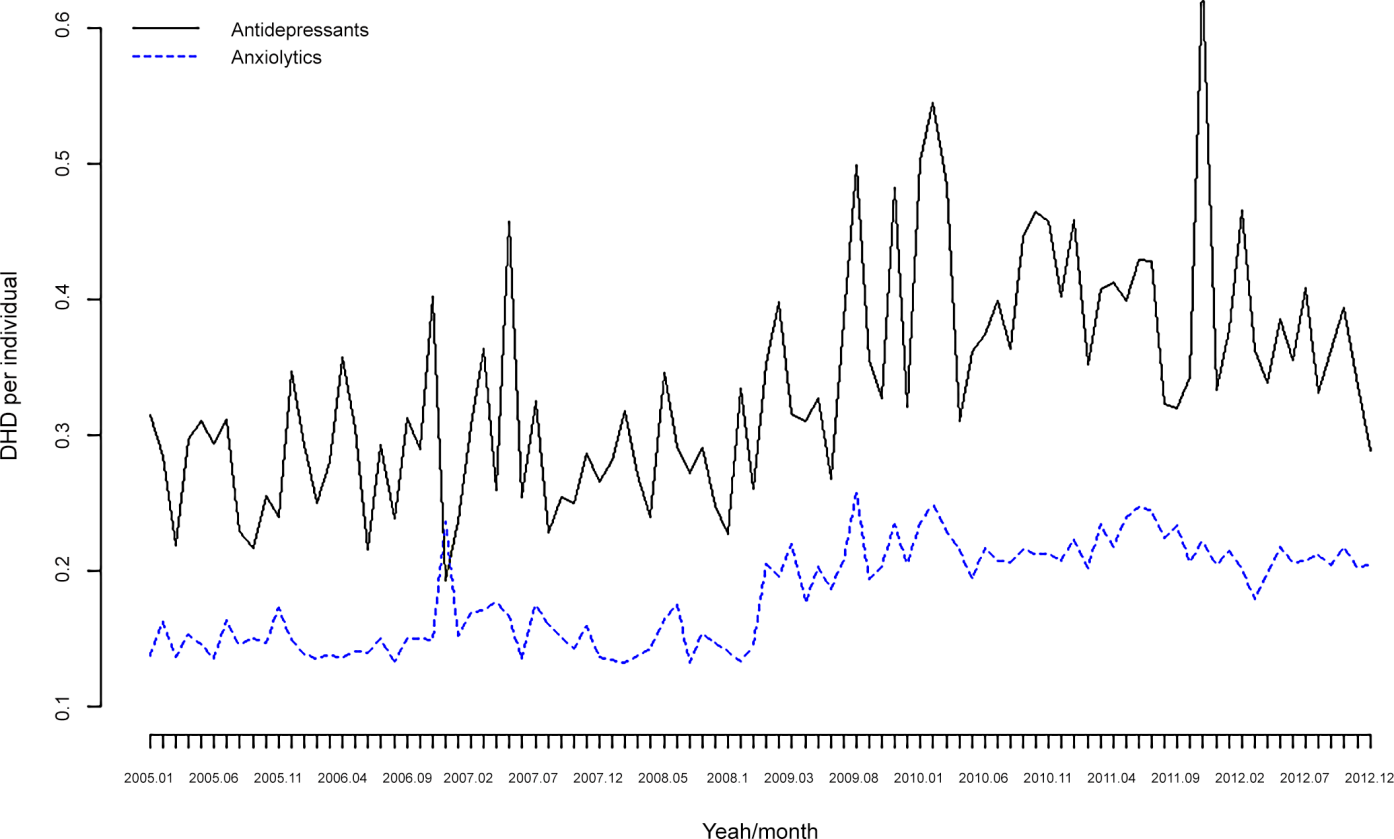
Consumption of psychotropic drugs. Antidepressants and anxiolytics. DDD per individual/month. Monthly data for the period 2005–2012.

In Tables 4 and 5 and Fig 3 and Fig 4, we show the consumption of psychotropic drugs stratified by anxiolytics and antidepressants. In the case of anxiolytics there was a statistically significant increase in the number of drugs per individual/month (Table 4 and Fig 3), whereas for DDD, there was an increase in both anxiolytics and antidepressants (Table 5), with antidepressants (see Fig 4) being far more important. Furthermore, the increases in the number of anxiolytics consumed in the sub-period 2009–2012 compared to 2005–2008 (see Table 4) are almost identical to the increases in the number of drugs (unstratified) shown in Table 2.

**Table 4:** Consumption of psychotropic drugs. Number of drugs per individual/month.

Number of drugs per individual/month	Anxiolytics			Antidepressants		
	2005–2008	2009–2012	p-value	2005–2008	2009–2012	p-value
**All**	1.163 (0.368)	1.201 (0.439)	0.001	1.146 (0.402)	1.145 (0.292)	0.906
** Not consumers before 2009**	---	1.203 (0.402)		---	1.157 (0.287)	---
** Always consumers**	1.163 (0.368)	1.200 (0.402)	0.002	1.146 (0.402)	1.140 (0.293)	0.731
**Sex**						
** Men**	1.172 (0.380)	1.216 (0.443)	0.003	1.186 (0.433)	1.132 (0.284)	0.118
** Women**	1.158 (0.363)	1.192 (0.389)	<0.001	1.133 (0.391)	1.143 (0.295)	0.603
**Age group**						
** 15–34 years**	1.171 (0.406)	1.208 (0.440)	0.106	1.147 (0.430)	1.161 (0.329)	0.784
** 35–44 years**	1.188 (0.405)	1.191 (0.413)	0.873	1.186 (0.547)	1.134 (0.296)	0.316
** 45–54 years**	1.169 (0.383)	1.207 (0.407)	0.046	1.112 (0.276)	1.148 (0.308)	0.280
** 55–64 years**	1.144 (0.338)	1.201 (0.426)	0.002	1.189 (0.397)	1.139 (0.269)	0.158
** 65–74 years**	1.153 (0.316)	1.177 (0.351)	0.078	1.138 (0.389)	1.138 (0.282)	0.996
** 75 years or older**	1.144 (0.339)	1.215 (0.408)	<0.001	1.077 (0.221)	1.128 (0.288)	0.120
**Probability of being unemployed**^1^						
** 1**^**st**^ **Quintile**	1.200 (0.440)	1.151 (0.407)	0.129	1.084 (0.246)	1.108 (0.268)	0.660
** 2**^**nd**^ **Quintile**	1.166 (0.390)	1.169 (0.381)	0.902	1.158 (0.385)	1.156 (0.330)	0.967
** 3**^**rd**^ **Quintile**	1.174 (0.368)	1.208 (0.448)	0.197	1.178 (0.409)	1.129 (0.265)	0.392
** 4**^**th**^ **Quintile**	1.154 (0.354)	1.214 (0.404)	0.001	1.214 (0.600)	1.134 (0.295)	0.143
** 5**^**th**^ **Quintile**	1.171 (0.390)	1.229 (0.434)	0.001	1.138 (0.343)	1.173 (0.297)	0.230
**Probability of being unemployed (long term)**^1^						
** 1**^**st**^ **Quintile**	1.201 (0.423)	1.120 (0.374)	0.024	1.142 (0.294)	1.086 (0.249)	0.404
** 2**^**nd**^ **Quintile**	1.178 (0.397)	1.171 (0.385)	0.664	1.172 (0.433)	1.143 (0.314)	0.570
** 3**^**rd**^ **Quintile**	1.161 (0.369)	1.185 (0.420)	0.365	1.120 (0.321)	1.153 (0.297)	0.488
** 4**^**th**^ **Quintile**	1.163 (0.368)	1.213 (0.415)	0.014	1.218 (0.617)	1.141 (0.311)	0.174
** 5**^**th**^ **Quintile**	1.165 (0.380)	1.234 (0.436)	<0.001	1.143 (0.346)	1.162 (0.283)	0.502

1 From least likely (first quintile) to most likely (fifth quintile) of being unemployed

Values represent mean (standard deviation)

**Table 5:** Consumption of psychotropic drugs. DDD per individual/month.

DDD per individual/month	Anxiolytics			Antidepressants		
	2005–2008	2009–2012	p-value	2005–2008	2009–2012	p-value
**All**	0.151 (0.368)	0.214 (0.548)	0.004	0.284 (0.511)	0.390 (0.745)	0.016
** Not consumers before 2009**	--	0.124 (0.295)	---	---	0.192 (0.324)	---
** Always consumers**	0.151 (0.368)	0.231 (0.583)	<0.001	0.284 (0.511)	0.415 (0.779)	0.001
**Sex**						
** Men**	0.119 (0.273)	0.187 (0.569)	<0.001	0.278 (0.477)	0.362 (0.956)	0.300
** Women**	0.167 (0.407)	0.228 (0.535)	<0.001	0.287 (0.522)	0.400 (0.658)	0.004
**Age group**						
** 15–34 years**	0.123 (0.394)	0.147 (0.664)	0.421	0.310 (0.585)	0.373 (1.207)	0.657
** 35–44 years**	0.132 (0.254)	0.169 (0.395)	0.026	0.246 (0.385)	0.297 (0.379)	0.343
** 45–54 years**	0.156 (0.427)	0.191 (0.422)	0.081	0.235 (0.322)	0.348 (0.439)	0.028
** 55–64 years**	0.171 (0.415)	0.235 (0.601)	0.012	0.390 (0.824)	0.394 (0.618)	0.966
** 65–74 years**	0.173 (0.402)	0.220 (0.442)	0.033	0.284 (0.403)	0.470 (0.848)	0.053
** 75 years or older**	0.153 (0.248)	0.289 (0.666)	<0.001	0.226 (0.245)	0.433 (0.846)	0.028
**Probability of being unemployed**^1^						
** 1**^**st**^ **Quintile**	0.100 (0.391)	0.096 (0.208)	0.872	0.322 (0.961)	0.178 (0.189)	0.311
** 2**^**nd**^ **Quintile**	0.116 (0.248)	0.123 (0.277)	0.530	0.293 (0.350)	0.205 (0.223)	0.064
** 3**^**rd**^ **Quintile**	0.129 (0.271)	0.136 (0.291)	0.197	0.231 (0.256)	0.223 (0.270)	0.870
** 4**^**th**^ **Quintile**	0.158 (0.470)	0.242 (0.742)	0.007	0.364 (0.823)	0.527 (1.258)	0.277
** 5**^**th**^ **Quintile**	0.177 (0.397)	0.279 (0.598)	<0.001	0.306 (0.482)	0.486 (0.616)	0.002
**Probability of being unemployed (long term)**^1^						
** 1**^**st**^ **Quintile**	0.096 (0.397)	0.060 (0.112)	0.154	0.337 (0.906)	0.137 (0.140)	0.272
** 2**^**nd**^ **Quintile**	0.119 (0.235)	0.119 (0.263)	0.995	0.329 (0.391)	0.205 (0.223)	0.013
** 3**^**rd**^ **Quintile**	0.119 (0.275)	0.153 (0.347)	0.099	0.170 (0.177)	0.243 (0.250)	0.068
** 4**^**th**^ **Quintile**	0.173 (0.501)	0.220 (0.655)	0.128	0.378 (0.855)	0.521 (1.269)	0.363
** 5**^**th**^ **Quintile**	0.172 (0.391)	0.275 (0.649)	<0.001	0.309 (0.478)	0.468 (0.600)	0.004

1 From least likely (first quintile) to most likely (fifth quintile) of being unemployed

Values represent mean (standard deviation)

In the two sub-periods some differences were observed in the behaviour of the DDD stratifying between anxiolytics and antidepressants (Table 5). Although for both men and women DDD increased for anxiolytics, increases for antidepressants only occurred for women. In addition, a statistically significant increase (p <0.05) in anxiolytic use was observed from 55 years onwards (apart from the 35–44 years age group) and from 75 years or older in the case of antidepressants (apart from the 45–54 age group). The behaviour of the probability of being unemployed (also being unemployed long term) is the same as the non-stratified behaviour between anxiolytics and antidepressants, albeit with the exception of the DDD of antidepressants where the increase in the fourth quintile was not statistically significant.

**Fig 5:**
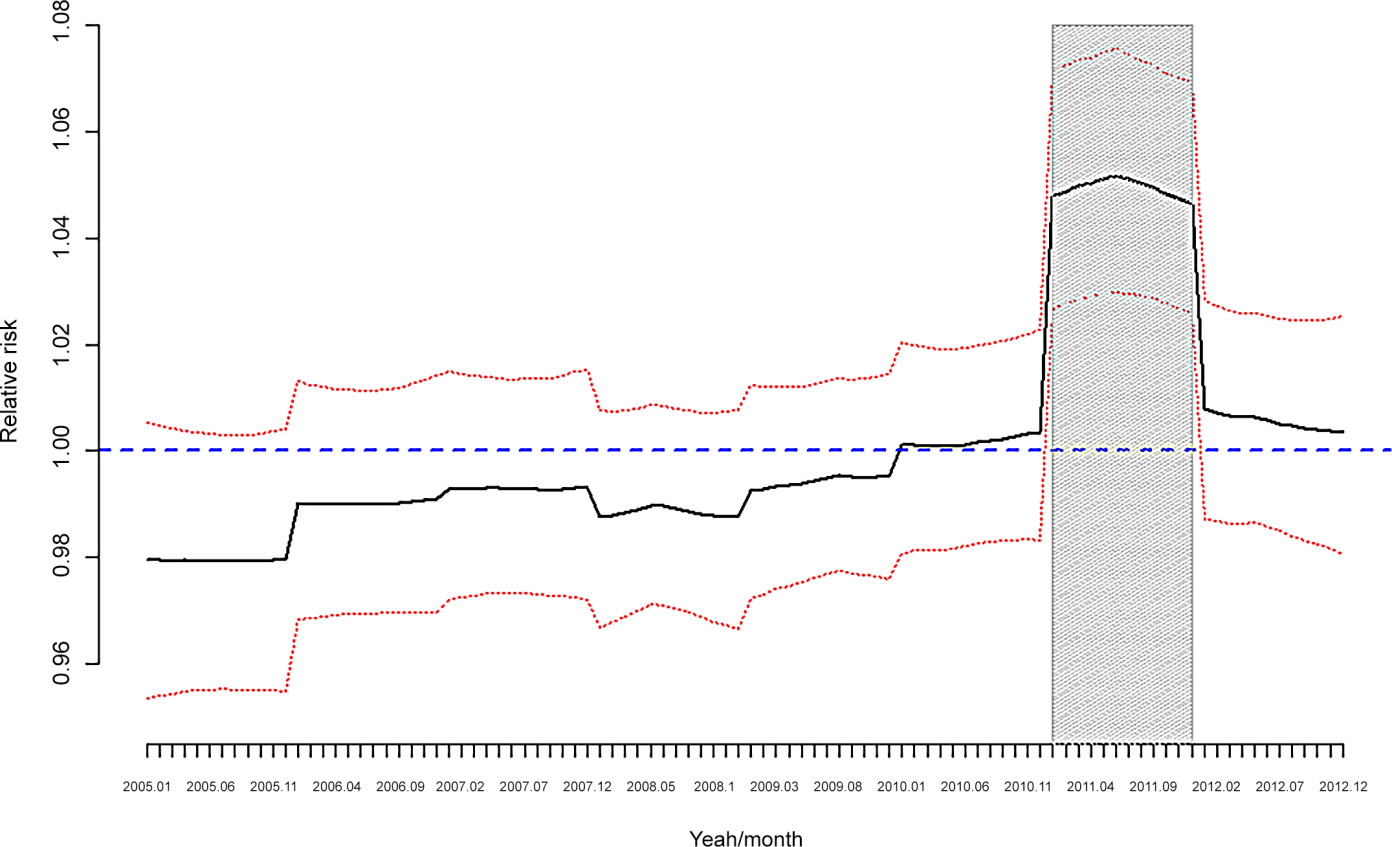
Number of drugs per person/month. **Monthly data for the period 2005–2012. Shaded** Statistically significant (the 95% credibility interval does not contain the unit).

**Fig 6:**
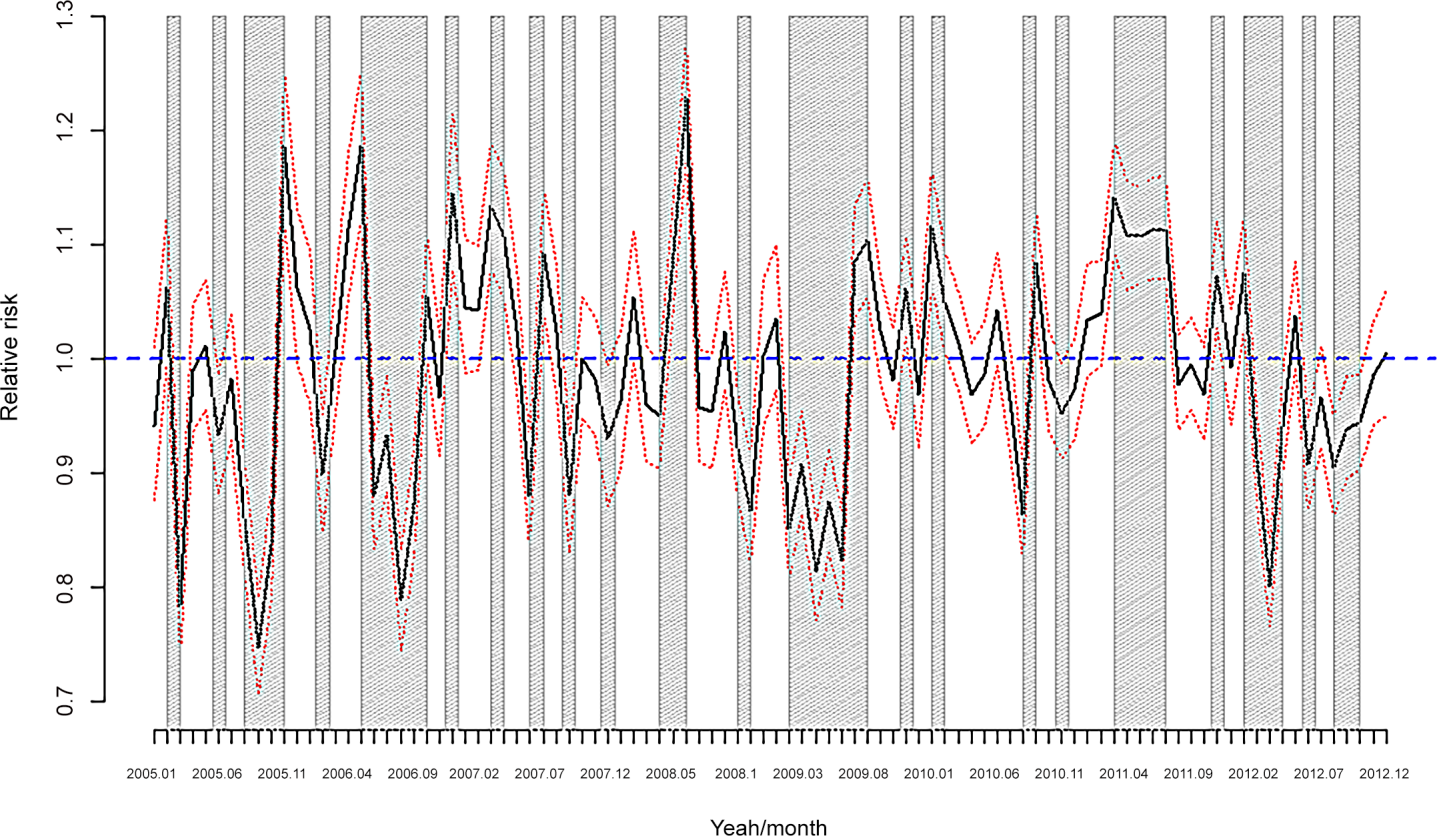
DDD per individual/month. **Monthly data for the period 2005–2012. Shaded** Statistically significant (the 95% credibility interval does not contain the unit).

**Fig 7:**
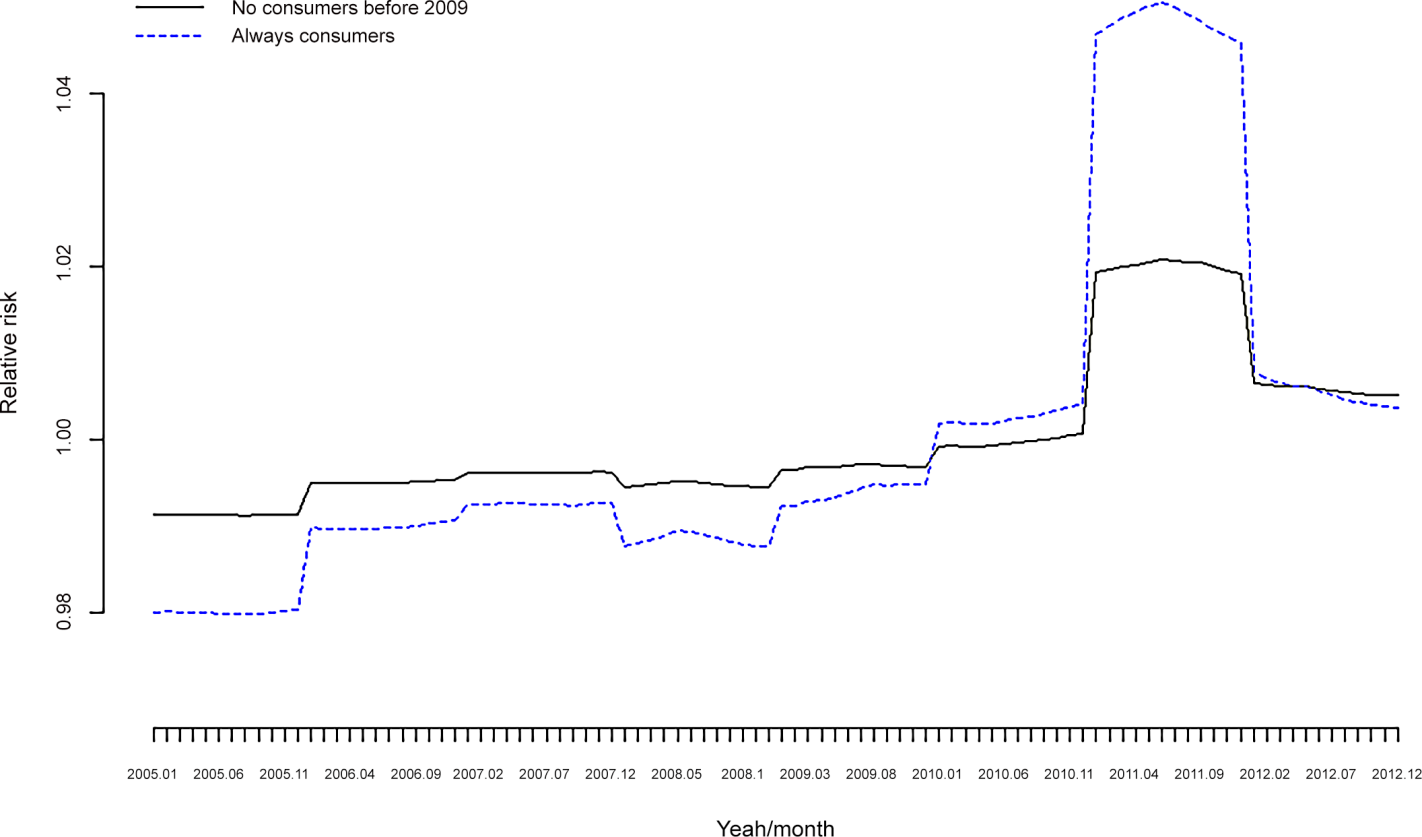
Number of drugs per person/month. Not consumers before 2009 vs. Always consumers.

**Fig 8:**
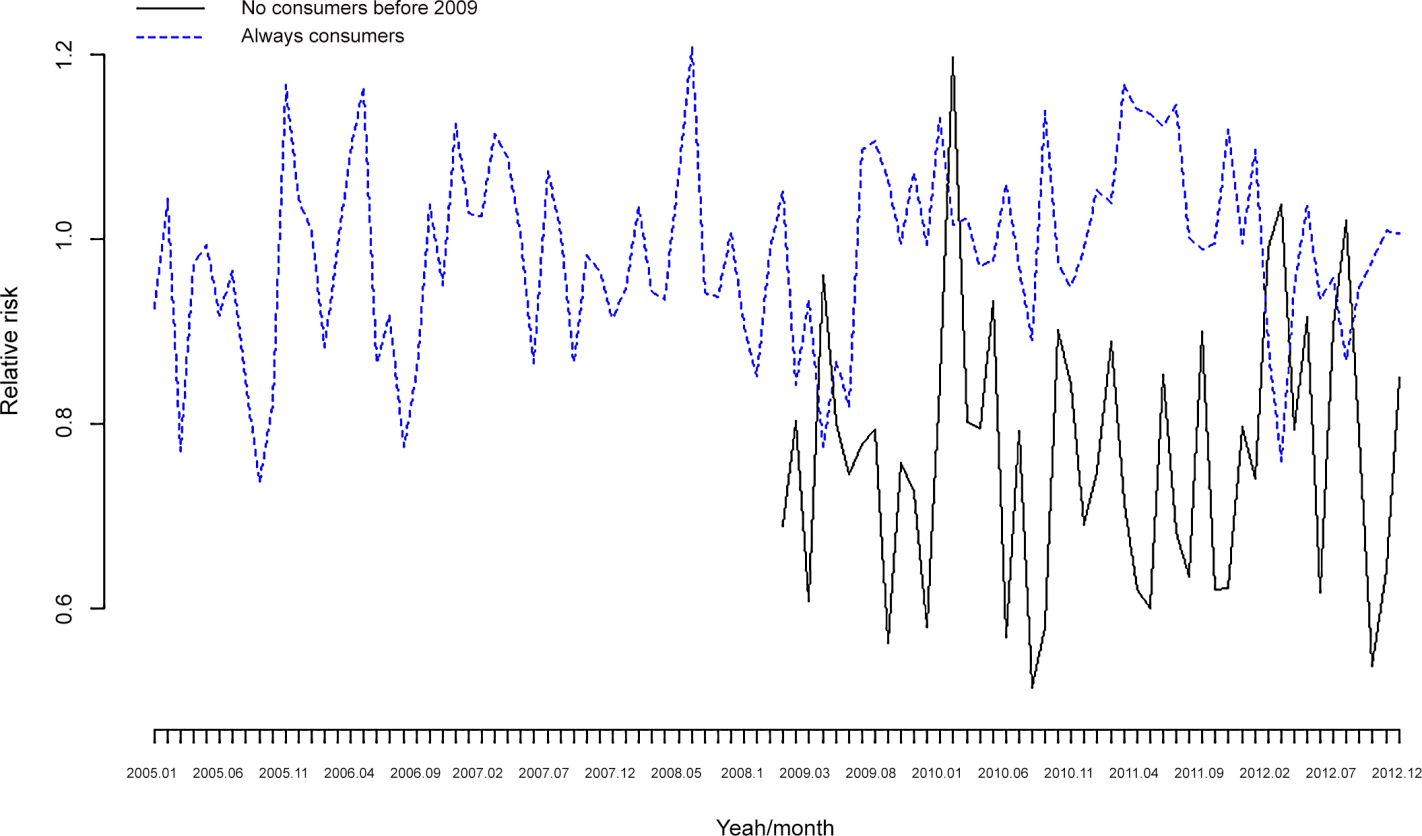
DDD per individual/month. Not consumers before 2009 vs. Always consumers.

The results of the estimation of the models are shown in Tables 6 and 7 and in Figs 5–8. The relative risks (RR) of consuming psychotropic drugs (the number of drugs per individual/month) in the period 2009–2012 compared to 2005–2008, were greater than unit, as much in the unstratified case as in the case of anxiolytics (Table 6). In fact, unstratified relative risks were very similar to the relative risks of anxiolytics. In both cases the increase in RR was statistically significant for the group of ‘always consumers’ (both genders), for individuals with metabolic syndrome (in this case the increase was statistically significant only for anxiolytics), for those individuals who were born in Spain, those who had family in the cohort, those older than 44 years, and individuals in the fourth and fifth quintile of the probability of being unemployed (and of being long-term unemployed).

**Table 6:** Multivariate analysis. Increase in the number of drugs (per individual/month). Relative risk 2009–2012 vs. 2005–2008.

	All	Anxiolytics	Antidepressants
**All**	1.13790*	1.11518*	0.83061
** Always consumers**	1.14427*	1.11563*	0.82856
**Sex**			
** Men**	1.20805*	1.11282*	0.83464
** Women**	1.10454*	1.11280*	0.82777
**Metabolic syndrome**			
** No**	1.13678	1.11553	0.83110
** Yes**	1.08450	1.37621*	0.77917 *
**Country of birth**			
** Spain**	1.12359*	1.11428*	0.82889
** Rest**	1.13570	1.10941	0.82603
**Family in the cohort?**			
** No**	1.13509	1.09090	0.82740
** Yes**	1.14649	1.10016*	0.84043
**Age group**			
** 15–34 years**	1.10840	1.11590	0.88521
** 35–44 years**	1.13267	1.11370*	0.88582
** 45–54 years**	1.13196*	1.11981*	0.88730
** 55–64 years**	1.13095*	1.10047*	0.81303
** 65–74 years**	1.13320*	1.18129*	0.84169
** 75 years or older**	1.25273*	1.17305*	0.83933
**Probability of being unemployed**^1^			
** 1**^**st**^ **Quintile**	1.09905	1.01940	1.04788
** 2**^**nd**^ **Quintile**	1.11111	1.03658	1.12099
** 3**^**rd**^ **Quintile**	1.04610	1.06363	1.03815
** 4**^**th**^ **Quintile**	1.16310*	1.18587*	0.71356
** 5**^**th**^ **Quintile**	1.13131*	1.13289*	0.79467
**Probability of being unemployed (long term)**^1^			
** 1**^**st**^ **Quintile**	1.00358	0.96687	1.02877
** 2**^**nd**^ **Quintile**	1.11425	1.05100	1.11294
** 3**^**rd**^ **Quintile**	1.07504	1.03222	0.96368
** 4**^**th**^ **Quintile**	1.12003*	1.17481*	0.69701
** 5**^**th**^ **Quintile**	1.13271*	1.14172*	0.81003

* The 95% credibility interval did not contain the unit (i.e. statistically significant)

1 From least likely (first quintile) to most likely (fifth quintile) of being unemployed

Models adjusted by sex, age group, country of birth, family in the cohort, metabolic syndrome, cancer, fear of having cancer, the quintiles of the probability of being unemployed (and also of being unemployed for over a year) and the monthly number of visits to a physician.

**Table 7:** Multivariate analysis. Increase in DDD. Relative risk 2009–2012 vs. 2005–2008.

	All	Anxiolytics	Antidepressants
**All**	1.07700 *	1.12287 *	1.05052*
** Always consumers**	1.09936 *	1.09396 *	1.12206*
**Sex**			
** Men**	1.05354*	1.07635*	1.03371
** Women**	1.15783*	1.10018*	1.21676*
**Metabolic syndrome**			
** No**	1.00054	1.02281	1.02030
** Yes**	1.06396*	1.03952	1.07521*
**Country of birth**			
** Spain**	1.01737	1.02773	1.03362
** Rest**	0.92125	1.03597	0.87934*
**Family in the cohort?**			
** No**	1.01782	1.02084	0.99849
** Yes**	1.01947	1.04287	1.01678
**Age group**			
** 15–34 years**	0.91622	0.99967	1.05418
** 35–44 years**	1.03448	1.10654*	1.01804
** 45–54 years**	1.05013*	1.01619	1.12270*
** 55–64 years**	0.98934	1.05351*	0.93835
** 65–74 years**	1.11873*	1.16324*	1.08540
** 75 years or older**	1.13744*	1.09011*	1.18293*
**Probability of being unemployed**^1^			
** 1**^**st**^ **Quintile**	0.91179	0.98108	0.75717
** 2**^**nd**^ **Quintile**	1.02956	1.05734	0.97420
** 3**^**rd**^ **Quintile**	1.02002	1.06328	0.97998
** 4**^**th**^ **Quintile**	1.02176*	1.14566*	1.01770
** 5**^**th**^ **Quintile**	1.12978*	1.19359*	1.08340 *
**Probability of being unemployed (long term)**^1^			
** 1**^**st**^ **Quintile**	0.91811	0.99635	0.83793
** 2**^**nd**^ **Quintile**	1.00019	1.00193	0.95041*
** 3**^**rd**^ **Quintile**	1.02327	1.07688	1.03004
** 4**^**th**^ **Quintile**	1.04605	1.04736	1.03329
** 5**^**th**^ **Quintile**	1.05158*	1.07350*	1.02607*

* The 95% credibility interval did not contain the unit (i.e. statistically significant)

1 From least likely (first quintile) to most likely (fifth quintile) of being unemployed

Models adjusted by sex, age group, country of birth, family in the cohort, metabolic syndrome, cancer, fear of having cancer, the quintiles of the probability of being unemployed (and also of being unemployed for over a year) and the monthly number of visits to a physician.

Fig 5 shows that the increase in RR occurred in 2011 and, although this increase occurred for both the ‘always consumers’ and ‘not consumers before 2009’ groups, the increase was much more important for the ‘always consumers’ (Fig 7).

The estimation results for the DDD are shown in Table 7 and in Fig 6 and Fig 8. While a statistically significant increase also occurred in RR 2009–2012 vs. 2005–2008 for anxiolytics and antidepressants (Table 7), this increase was not as evident as it was in the number of drugs (Fig 6). Although the results were similar to anxiolytics and antidepressants, there were some differences (Table 7). For example, the increase in RR for antidepressants was (statistically) significant for women, but it was (statistically) significant for both men and women for anxiolytics. Again in the case of antidepressants, the increase in RR was (statistically) significant for individuals with metabolic syndrome (but not for anxiolytics), for individuals aged 75 years or older, for the 45–54 year old group and for individuals located in the fifth quintile of the probability of being unemployed. Meanwhile for anxiolytics the older than 55 years group (in this case 35 individuals 44 years old) and the fourth and fifth quintile were (statistically) significant.

When the ‘not consumers before 2009’ are compared to the ‘always consumers’ group (Fig 8), the relative risks of the ‘always consumers’ are always higher, however, there appears to be an increasing trend from 2011 onwards in the ‘not consumers before 2009’, which is not observed in the ‘always consumers’.

## Discussion

By comparing the period 2005–2008 prior to the economic crisis with the crisis period itself, we found there was in fact an increase in psychotropic drug use in the general population in a semi-urban region in Catalonia, Spain.

Although this increase was quite general, there are certain peculiarities that should be noted. First, the increase in consumption was significantly higher in individuals who had been using psychotropic drugs prior to 2009. Second, the increase in use occurred in those most likely to be unemployed (i.e. located at the fourth and fifth quintile of the probability of being unemployed, in all unemployed and in long-term unemployed). Third, the increase was more pronounced when the consumption was measured by the number of drugs (per individual/month) rather than using the DDD (also per individual/month). Fourth, at least for the number of drugs, the increase occurred in 2011 coinciding with the year in which the major effects of the crisis in Spain manifested themselves.

We can assume that the number of drugs would provide an idea of the severity of the mental health disorder, as various treatments are tested for each patient or combined to obtain a positive response, and that the DDD would indicate the intensity of the treatment. Hence, after the economic recession began in 2009 there was an increase in the severity rather than the intensity of mental health disorders in those individuals who had already been suffering from mental illness before the crisis. This increase occurred for individuals most likely to be unemployed, and its severity was accentuated in the harshest year of the economic crisis.

There were also differences (with respect to the pre-crisis period) in the increased consumption of both anxiolytics and antidepressants. Thus, while there was an increase of only severity (measured by the number of drugs) in the case of anxiolytics, there was an increase in both severity and intensity (determined by DDD) in the case of antidepressants.

Finally, the increase in consumption (again, with respect to the pre-crisis period) was not uniform for all population groups. There was an increase in both the severity and the intensity in the case of anxiolytics for individuals 45–55 years or older, and also in intensity for individuals 75 years or older; albeit only in the case of antidepressants.

Increased consumption of antidepressants in individuals 75 years or older may be due to at least two factors: the anxiety generated by the fact that they are the ones who economically sustain their (now adult) children; and/or that the drugs are actually for their (adult) children and they are taking advantage of their pensioner status to save on the cost of that drug.

There are several findings that could corroborate these two arguments. In the case of the first argument, while with anxiolytics the increase in severity and intensity occurred in both sexes, in the case of antidepressants there was only an increase in intensity and only for women. Older women (i.e. grandmothers) are those who, to a greater extent, hold the family together in these times of economic crisis.

Along the lines of the second argument, there was also an increase in intensity for the 35 to 44 age group in the case of anxiolytics, and for the 45–54 age group in the case of antidepressants. In addition, and perhaps supporting both arguments, in the case of anxiolytics there was an increase in severity in the 2009–2012 period for those individuals who had family members in the cohort. Again, this would mean more support than others who did not have family members within the cohort.

The evidence of the effects of the Great Recession on psychotropic drug consumption is sparse. A search for articles published in the last five years using the terms 'psychotropic', 'psychotropic drugs', 'antidepressant', 'antipsychotics', 'hypnotics’ and 'anxiolytics', combined with ‘financial crisis’, ‘recession’, ‘economic recession’, ‘economic crisis’, and ‘economic downturn’ in PubMed and Embase between March and November 2014, provided the grand total of a mere twenty-one articles. Only seven of the articles provided quantitative, rather than purely descriptive, evidence of the relationship between the consumption of psychotropic drugs and the current economic recession [23,27,28,49–51].

In five of these seven studies, the unit of analysis is ecological and in the other two, individual. The only two studies we found on an individual level showed an increase in the consumption of psychotropic drugs in the US during the recession [50,51]. Using data from several waves of the RAND HRS, a nationally representative longitudinal survey from the US of more than 22,000 people over the age of 50, McInerney *et al*. found that the stock market crash had caused an increase in the use of antidepressant drugs (as well as feelings of depression), and that these effects were greatest among respondents with high levels of stock holdings prior to the crash [50]. Meanwhile Chen and Dagher using the US nationally representative dataset from the Medical Expenditure Panel Survey of 2000–2009, found that prescription drug use increased significantly during the economic recession, although health visits for mental health disorders decreased during that same period [51].

The evidence in ecological studies is controversial. In fact, only Bradford and Lastrapes, who use monthly data from the National Ambulatory Medical Care Survey (NAMCS) of non-federal employed office-based physicians aggregated by the US census regions, find that prescriptions for antidepressants and anti-anxiety drugs increase when unemployment rises and employment falls in the US, albeit only in the Northwest [23]. In addition, they find weaker evidence of a counter-cyclical behaviour of doctor visits culminating in mental health diagnosis. Ilyas and Moncrieff, employing data from prescriptions in the UK between 1998 and 2010, find an increase in antidepressant and antipsychotic prescriptions but no change in hypnotic and anxiolytic prescriptions [49]. However, unlike Wise [25], no change seemed to occur in the trend of prescriptions for antidepressants and antipsychotics from the beginning of economic recession.

With the exception of Bradford and Lastrapes [23], our findings do not agree with those of the ecological studies. However, critics of ecological studies point to the inability to make inferences on the individual level. In fact, individual responses to the recession need not be equal to the ‘average’ aggregate response [23]. Closely related to this limitation is the inability to control for confounding on the individual level. As noted earlier, ecological measures have even been shown to have a much smaller effect than the measures obtained for mental health indicators on an individual level [12,14].

However, our findings are consistent with those found in only two of the recently published studies on the individual level of the effects of the economic recession on the consumption of psychotropic drugs and mental health [50,51]. Thus, in Chen and Dagher prescription drug use increased significantly during the economic recession [51]. In the same vein, McInerney *et al*. found that people over the age of 50 increased their use of antidepressant drugs [50]. It should be noted, however, that these two studies may contain some methodological flaws. Despite both using a panel data design, neither control the presence of individual heterogeneity or temporal heterogeneity. The most that both studies do is to allow the recession to have heterogeneous effects albeit not for individuals, but rather for different groups of individuals (individuals with and without stock portfolios prior to the financial crisis, in McInerney *et al*. [50]; ethnic groups: Latino, African American and other, in Chen and Dagher [51]).

Clearly, our results could have been influenced by the differing effects of the Great Recession in the region we focussed on, although these would be no different to the rest of Spain.

The Great Recession in Spain has some rather distinctive aspects to it. Firstly, it started later in Spain than in other countries as the Spanish economy went into recession in the first quarter of 2009; after the GDP had fallen for two consecutive quarters. The Spanish economy did emerge from this first recession in the first quarter of 2010, when the GDP showed positive growth rates, only to slip back into recession in the second quarter of 2011 (double dip) and not to come out of it again until the third quarter of 2013. Secondly, the recession in Spain has been much more severe than in other parts of the world. For instance, in the second quarter of 2007 unemployment had dropped to a record low of 7.95% (note how high it was, despite being the lowest since 1975). However, during the recession it soared to over 20%, climbing to a record high of 27.16% in the first quarter of 2013. Furthermore, the unemployment rate among youth (16–25 year olds) reached 57.2%.

On the other hand, it was not until May 2010 that the Spanish government adopted fiscal austerity measures to fight the crisis. Public spending on health went from its previous steady growth, (a median of 7.5% per annum from 2003 to 2009), to fall by 3% per annum from 2010 onwards (at a median). Public spending on education went from increasing 6.68% per annum to decreasing 3.16% per annum from 2010 onwards (all medians). The largest drop (nearly 10%) in public spending on health and education was in 2011 [52].

If, as Karanikolos *et al*. claim, interaction between fiscal austerity, economic shocks and weak social protection more than the recession itself pose a risk to health [53], then in Spain the effects of the Great Recession on health in general, and mental health in particular, should have manifested themselves from 2010. Following this argument, our results (Figs 5 and 7) show that the difference in the number of drugs (per person and in DDD) is much more evident from the latter stage of 2010 onwards.

The main limitation to this study was not having any direct measure of the socio-economic status of the individuals in the cohort. This has prevented us, firstly, from assessing the variation in the consumption of psychotropic drugs by employed individuals who had seen their salaries reduced and/or their working conditions worsen as a result of the crisis. Secondly, as we were not able to measure exposure on the individual level we cannot attribute the increase in consumption of psychotropic drugs to the economic recession, but can simply argue that the increase occurred after 2009, and to be precise in 2011, the worst year of the crisis in Spain.

Although family level mental health variables influence mental health utilization, we did not have that information.

In addition to the consumption of prescribed psychotropic drugs, we could have used violence, suicides, self-harm and related indicators as response variables. However, in Spain is not possible to find information on these variables on an individual level as it is only available on an ecological level. Moreover, the authorities do not provide information, including aggregate, for municipalities with fewer than 10,000 inhabitants (see, e.g. [54]). That is to say, we would have had information (and only aggregate) for only one single municipality out of the 144.

Our contribution to the existing literature is threefold. Firstly from a methodological point of view and in contrast to the earlier literature which, like us, use a panel data on an individual level, we specified a generalized linear mixed model, combining ‘selection on observables’ variables such as (propensity scoring) matching and ‘selection on unobservables’ such as (random coefficient) panel data model methods, and we performed inferences using a Bayesian framework. Besides controlling for individual confounders, we explicitly take into account both individual and temporal heterogeneity. Furthermore, we allowed (some of the) coefficients to be different for, mainly, individual and month. This enabled us to assess whether the economic recession and/or the course of the recession had different effects on individuals. Secondly, from an empirical point of view, besides assessing antidepressants and anxiolytics separately, we distinguish between the severity of the mental disorder (measured by the number of drugs) and the intensity of the treatment (measured by the defined daily dose (DDD)). We found that, with respect to the period prior to the economic crisis (2005–2008), there was an increase in the consumption of prescribed psychotropic drugs. However, and this is our third contribution, this increase was in the severity rather than the intensity of the mental health illness of those individuals who had already been diagnosed with disorders before the crisis. This increase occurred in individuals most likely to be unemployed, and severity was accentuated in the bleakest year of the economic crisis.

## Supporting Information

S1 Appendix Method of the estimation of the probability of an individual being unemployed. (DOC)
