# Supplementary material for: Effects of the Financial Crisis on Psychotropic Drug Consumption in a Cohort from a Semi-Urban Region in Catalonia, Spain
Source: PLoS One. 2016 Feb 12;11(2):e0148594. doi: 10.1371/journal.pone.0148594 (PMC4752355; doi:10.1371/journal.pone.0148594)
Supplement: S1 Appendix — (DOC) [file pone.0148594.s001.doc]

**S1 Appendix. Method of the estimation of the probability of an individual being unemployed**

Using data from the Spanish and the Catalan Health Surveys (ENSE and ESCA, respectively) corresponding to 2006 and 2011 (excluding those individuals under 16 and over 65 who were inactive and therefore with a zero probability of being unemployed), the probability of an individual being unemployed (or unemployed for over a year) was estimated using the following GLM with a binomial response (i.e. logistic regression):

$$\Pr ob\left(Y_{i}=1\right)=E\left(Y_{i}\right)=\mu_{i}$$

$$\begin{matrix} \log\left(\frac{\mu_{i}}{1-\mu_{i}}\right)=\beta_{0i}+\beta_{1}Sex_{i}+\beta_{2}Age+\beta_{3}HTA_{i}+\beta_{4}DM2_{i}+\beta_{5}Obesity_{i}+\beta_{6}HDL_{i}+\beta_{7}TG_{t}+ \\ \beta_{8}Doctor\_Visits_{i}+\beta_{9}Birth\_Country_{i}+\beta_{10}\mathrm{Re}gion_{i}+\beta_{11}Stratum \end{matrix}$$

where Y denoted the event of being unemployed (or unemployed for over a year).

As explanatory variables the model included: sex, age (introduced here as a continuous variable), medical conditions (which were self-declared): elevated blood pressure (HTA), elevated fasting plasma glucose or diabetes mellitus type II (DM2), obesity (BMI > 30 kg/m^2^), low high-density cholesterol (HDL) levels, high serum triglycerides (TG), the number of doctor visits (Doctor_Visits) an individual made in the past twelve months (in the ESCA) or in the past three months (in the ENSE), the country of birth of the individual (Birth_Country), the ‘county’ of residence^a^ (Region) (only in the ESCA), and the size of the municipality of residence (Stratum) (only in the ENSE).

For each of the periods considered (2006 and 2011) we estimated two vectors of probabilities (one corresponding to ESCA and the other to ENSE): that an individual, with the characteristics defined by the explanatory variables, was unemployed (or unemployed for over a year). Then, using the estimated linear predictor (i.e. the right hand side of the equation), we predicted these two sets of probabilities (for being unemployed and for being unemployed for over a year) that would correspond to the individuals in the cohort. To do this, we first divided the cohort into two sub-periods: 2005-2009 and 2010-2012. We used the first sub-period to predict the probabilities associated with the ESCA and ENSE 2006 and the second to predict those associated with the ESCA and ENSE 2011. We assigned a zero probability to individuals under 16 and to those over 65 or who were older than 65 during the study period. Next we stacked the probabilities corresponding to the two sub-periods and estimated the following GLM with Gaussian response (i.e. a linear regression):

$$E\left(\Pr ob\_ESCA_{it}\right)=\beta_{0i}+\beta_{1}\Pr ob\_ENSE_{it}+\beta_{2}Unempl\_rate_{it}$$

where Prob_ESCA denoted the predicted probability of being unemployed (or unemployed for over a year) corresponding to ESCA, for the individual i in the month t; Prob_ENSE the predicted probability corresponding to ENSE; and Unempl_rate the unemployment rate in the municipality where individual i resided, for the sex of individual i, and for the year corresponding to the month t (these rates were obtained from IDESCAT, *‘Institut d’Estadística de Catalunya’* ([www.idescat.cat](http://www.idescat.cat/)).

Thus, we calibrated the probability of being unemployed (and unemployed for over a year), finally obtaining a single time-varying variable per individual.
